# Factors associated with antimicrobial resistant enterococci in Canadian beef cattle: A scoping review

**DOI:** 10.3389/fvets.2023.1155772

**Published:** 2023-04-20

**Authors:** Kayla M. Strong, Kaitlin L. Marasco, Jesse Invik, Heather Ganshorn, Richard J. Reid-Smith, Cheryl L. Waldner, Simon J. G. Otto, John P. Kastelic, Sylvia L. Checkley

**Affiliations:** ^1^Faculty of Veterinary Medicine, University of Calgary, Calgary, AB, Canada; ^2^AMR—One Health Consortium, Calgary, AB, Canada; ^3^Libraries and Cultural Resources, University of Calgary, Calgary, AB, Canada; ^4^Centre for Food-borne, Environmental and Zoonotic Infectious Diseases, Public Health Agency of Canada, Guelph, ON, Canada; ^5^Department of Population Medicine, University of Guelph, Guelph, ON, Canada; ^6^Western College of Veterinary Medicine, University of Saskatchewan, Saskatoon, SK, Canada; ^7^HEAT-AMR (Human-Environment-Animal Transdisciplinary Antimicrobial Resistance) Research Group, School of Public Health, University of Alberta, Edmonton, AB, Canada

**Keywords:** antimicrobial resistance, *Enterococcus* spp., beef, cattle, scoping review

## Abstract

**Introduction:**

Antimicrobial resistance (AMR) is a global health concern, occurring when bacteria evolve to render antimicrobials no longer effective. Antimicrobials have important roles in beef production; however, the potential to introduce AMR to people through beef products is a concern. This scoping review identifies factors associated with changes in the prevalence of antimicrobial-resistant *Enterococcus* spp. applicable to the Canadian farm-to-fork beef continuum.

**Methods:**

Five databases (MEDLINE, BIOSIS, Web of Science, Embase, and CAB Abstracts) were searched for articles published from January 1984 to March 2022, using *a priori* inclusion criteria. Peer-reviewed articles were included if they met all the following criteria: written in English, applicable to the Canadian beef production context, primary research, *in vivo* research, describing an intervention or exposure, and specific to *Enterococcus* spp.

**Results:**

Out of 804 screened articles, 26 were selected for inclusion. The included articles discussed 37 factors potentially associated with AMR in enterococci, with multiple articles discussing at least two of the same factors. Factors discussed included antimicrobial administration (*n* = 16), raised without antimicrobials (*n* = 6), metal supplementation (*n* = 4), probiotics supplementation (*n* = 3), pen environment (*n* = 2), essential oil supplementation (*n* = 1), grass feeding (*n* = 1), therapeutic versus subtherapeutic antimicrobial use (*n* = 1), feeding wet distiller grains with solubles (*n* = 1), nutritional supplementation (*n* = 1) and processing plant type (*n* = 1). Results were included irrespective of their quality of evidence.

**Discussion:**

Comparability issues arising throughout the review process were related to data aggregation, hierarchical structures, study design, and inconsistent data reporting. Findings from articles were often temporally specific in that resistance was associated with AMR outcomes at sampling times closer to exposure compared to studies that sampled at longer intervals after exposure. Resistance was often nuanced to unique gene and phenotypic resistance patterns that varied with species of enterococci. Intrinsic resistance and interpretation of minimum inhibitory concentration varied greatly among enterococcal species, highlighting the importance of caution when comparing articles and generalizing findings.

**Systematic Review Registration:**

[http://hdl.handle.net/1880/113592]

## 1. Introduction

In 2015, the World Health Organization stated there was a “global consensus that antimicrobial resistance poses a profound threat to human health” and released a call to action to address antimicrobial resistance (AMR) (1). Antimicrobial resistance is a quintessential One Health problem interwoven within and across human, animal, and environmental health. Antimicrobial resistance can spread within and between various interconnecting continuums; however, the means and extent of resistance transmission and maintenance are not fully elucidated. The epidemiology of AMR is complex. Antimicrobial resistant enterococci and genetic material coding for AMR can undergo multi-directional transmission among people, animals and the environment, related to numerous factors that influence development of resistance, likelihood of transmission, and/or likelihood of colonization in host and/or reservoir. The environment remains a largely unquantified reservoir of AMR. Antimicrobial-resistant bacteria and genetic material coding for AMR could be transmitted to people in various ways along the beef production continuum, including: direct contact between livestock and humans; environmental contamination by sewage or waste contaminating water, food or other fomites; and contamination during slaughter, processing, food handling, or home preparation (2). In 2016, the Food and Agriculture Organization (FAO) released a call for further research to address knowledge gaps in livestock-driven AMR (3) that was echoed in academic literature (4).

Canadian beef producers use antimicrobials to prevent and treat diseases. For example, tetracyclines and macrolides are commonly used in beef production in Canada (5). Studies examining tetracycline and macrolide resistance trends have reported varying extents of bacterial resistance in enteric bacteria (2, 6, 7). An Alberta enterococci study identified 59% of bovine fecal *Enterococcus hirae* resistant to tetracycline and 33% resistant to macrolides (2) based on Clinical and Laboratory Standards Institute (CLSI) interpretive criteria.

Antimicrobial resistance is a concern for the beef industry due to increasing AMR in common bacteria (e.g., *Mannheimia haemolytica*) coupled with infrequent commercialization of new antimicrobials. Consequently, available antimicrobial options may become limited, especially for metaphylaxis (8, 9). In response to these concerns, government and industry have launched surveillance programs across the production continuum to monitor resistance trends (10).

*Enterococcus* spp. are commensal bacteria present in the gastrointestinal flora of humans and livestock, comprising up to 1% of intestinal microbiota in adults (11). Enterococci are becoming an important multi-drug-resistant nosocomial pathogen associated with human infections, including endocarditis, bacteremia, and urinary tract infections (12, 13). *Enterococcus* spp. have intrinsic resistance to most cephalosporins and semi-synthetic penicillins and to low concentrations of penicillin and ampicillin (14). Enterococci are also intrinsically resistant (*in vivo*) to clindamycin, trimethoprim-sulfamethoxazole and low concentrations of aminoglycosides (14, 15). Aminoglycosides can be used for treatment when used with a combination of high concentrations of penicillin (14, 15). In addition, various species of enterococci have varying intrinsic resistance. For example, *Enterococcus faecalis* is naturally resistant to quinupristin-dalfopristin whereas *Enterococcus faecium* is not (14). *Enterococcus gallinarum* and *Enterococcus casseliflavus* are intrinsically resistant to low concentrations of vancomycin, although other species of *Enterococcus* are not (16).

Enterococci can also acquire resistance to most antimicrobial classes (including higher concentrations of penicillin and ampicillin) and can transfer mobile genetic elements to other bacteria. More than 62 species of enterococci are associated with infections in numerous hosts, with variations of intrinsic resistance and likely differing niches within the microbiome (17, 18). The population structure of *Enterococcus* spp. within the mammalian gastrointestinal tract is influenced by host species, host age, diet and environmental stress, season, portion of the gastrointestinal tract, and isolates studied (19). Given their location in the mammalian gut, enterococci are exposed to numerous other bacteria. Consequently, enterococci can efficiently accumulate resistance genes from other bacterial species, making them useful for assessing AMR in the gastrointestinal microbiome. Therefore, they are often used as a Gram-positive indicator in AMR surveillance. Of specific concern are human hospital-acquired vancomycin-resistant enterococcal infections; they are associated with higher mortality, extended patient stays, increased risk of re-admission, and higher treatment costs (20).

A scoping review may be used to describe available literature (including the volume and complexion of publications), evaluate the feasibility of a meta-analysis, or identify knowledge gaps in available literature (21). No published scoping reviews were identified that addressed associations between antimicrobial resistant enterococci and factors along the beef production continuum. In this context, factors are defined as modifiable actions or interventions that could be associated with an increase or decrease in antimicrobial-resistant enterococci or related resistance genes. A notable systematic review considered the effects of macrolide use on enteric bacteria and was scrutinized in the development of this scoping review (22).

The objectives of this scoping review were to: identify articles that investigate factors potentially associated with a change in the prevalence of AMR in *Enterococcus* spp. during various production stages applicable to the Canadian beef cattle industry; collate factors during beef cattle production (cow-calf and feedlot operations), processing, and retail markets; and identify the existing range of evidence and knowledge gaps in the literature.

## 2. Methods

This scoping review followed Arksey and O’Malley’s framework (23) and is reported using the PRISMA-ScR (Preferred Reporting Items for Systematic reviews and Meta-Analyses extension for Scoping Reviews) checklist (24). The scoping review used a population, concept and context framework when developing the question (25). The population in question was beef cattle and beef products; the concept examined was antimicrobial-resistant *Enterococcus* spp.; and the context was from cow–calf operations to retail meats.

### 2.1. Scoping review protocol

A search strategy was developed *a priori* following consultation with a multidisciplinary team that included a health science librarian, biostatisticians, geographers, veterinarians, and epidemiologists. The protocol for this review was registered on PRISM: University of Calgary Digital Repository (26). To identify articles published from January 1984 to the search date of March 2022, a search was done on the following databases: MEDLINE (Ovid platform), BIOSIS Previews (Web of Science platform), Web of Science Core Collection (Science Citation Index and Emerging Sources Citation Index), Embase (Ovid platform), and CAB Abstracts (EBSCO platform). The CAB Abstracts search is provided in Supplementary Figure S1; this search was translated to the syntax and vocabulary of the other databases. Covidence systematic review software (Veritas Health Innovation, Melbourne, Australia) was used to support abstract and full-text screening. The title and abstract screening and full-text screening were subjected to a double-blinded process. Each article was reviewed by a minimum of two reviewers (KS and JI), with a third reviewer (SC) introduced in instances of conflict. Articles were initially screened by title and abstract; those that appeared to meet inclusion criteria had full-text screening and were included or excluded, based on the following inclusion criteria:

1. Articles must be written in English and published after 1984, coinciding with formal acceptance of the genus *Enterococcus* (17).
2. Article factors must apply to the Canadian beef production context. Depending on the intervention described, the population of interest must have antimicrobial stewardship practices and animal production policies comparable to Canadian beef production. This would include similarities in legislation related to antimicrobial use and residues, plus similar production and management including cow-calf and feedlot production.
3. Articles must present peer-reviewed primary research; therefore, reviews, opinion articles, editorials, theses, and conference abstracts were not eligible. Consequently, research findings were evidence-based, peer-reviewed, and not replicated.
4. Articles must present *in vivo* studies. To ensure that the review was evidence-based and to improve generalizability in beef cattle production, only field trials were evaluated.
5. Articles must have a comparison of the effects of a factor that measured AMR in *Enterococcus* spp.

The decision guide used by the review team is provided (Supplementary S2). Cohen’s Kappa was used to assess the reproducibility of the screening process. Data extracted from articles were entered using an integrated Covidence extraction form. Detailed instructions were developed to guide the extraction process (Supplementary S3). Following extraction, data were exported and stored in a standardized Microsoft Excel spreadsheet.

## 3. Results

### 3.1. Screening process

**Figure 1:**
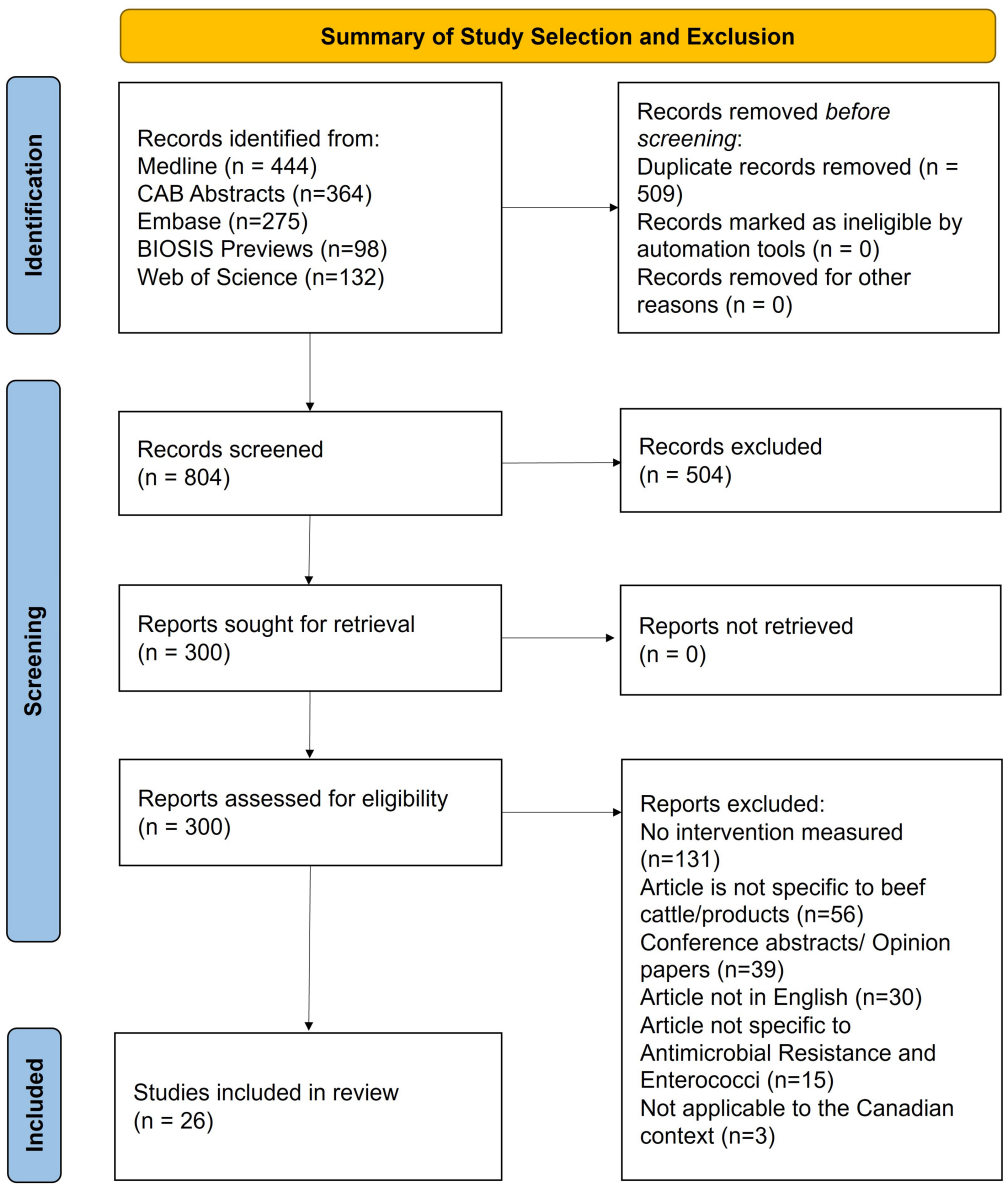
PRISMA-ScR flow diagram of the stages of article selection for inclusion in the Scoping review of reported factors associated with antimicrobial resistant enterococci in Canadian beef cattle.

Given the selection parameters, 1,313 studies were imported for screening and 509 duplicates were removed. A total of 804 articles were screened and 26 were selected, with characteristics summarized in Table 1. The article screening process is detailed in Figure 1. Cohen’s Kappa was 0.71 for the title and abstract and 0.66 for full-text screening, considered substantial agreement (53).

**Table 1:** Summary of the attributes of 26 articles included in the scoping review of reported factors associated with antimicrobial resistant enterococci in Canadian beef cattle.

First author and year of publication	Study design	Location	Beef production stage	Age of cattle (if applicable)	Exposure or intervention studied	Sample collection site	Compound administered (if applicable)
Agga (2016) (27)	Cross-sectional study	Nebraska, United States	Farm	Cows	Antimicrobial administration	Rectum	Ceftiofur
Amachawadi (2013) (28)	Randomized controlled trial	Kansas, United States	Feedlot	Yearlings	Metal supplementation	Pen floor	Copper
Amachawadi (2015) (29)	Randomized controlled trial	Kansas, United States	Feedlot	Fat cattle preslaughter	Metal and antimicrobial administration	Pen floor	Copper and Tylosin
Beukers (2015) (30)	Randomized controlled trial	Alberta, Canada	Feedlot	Fall placed calves	Antimicrobial administration	Rectum	Tylosin
Chan (2008) (31)	Cross-sectional study	Rhode Island, United States	Retail	Not applicable	“All natural” labelling	Retail	Not applicable
Davedow (2020) (32)	Randomized controlled trial	Alberta, Canada	Feedlot	Yearlings	Antimicrobial administration	Pen floor	Tylosin
Edrington (2014) (33)	Randomized controlled trial	Texas, United States	Feedlot	Fall-placed calves	Antimicrobial administration	Rectum	Virginiamycin
Halleran (2021) (34)	Non-randomized trial	North Carolina, United States	Feedlot	Fall placed calves	Antimicrobial administration	Rectum	Danofloxacin
Halleran (2021) (35)	Non-randomized trial.	North Carolina, United States	Feedlot	Fall placed calves	Antimicrobial administration	Rectum	Florfenicol
Hershberger (2005) (36)	Cross-sectional study	United States, multiple states	Farm	Cows and pre-weaned calves at cow-calf operations	Antimicrobial administration	Rectum	Not specified
Innes (2021) (37)	Cross-sectional study	United States, multiple states	Retail	Not applicable	USDA-Certified Organic labeling Processing plant type	Retail	Not applicable
Jacob (2008) (38)	Randomized controlled trial	Kansas, United States	Feedlot	Yearling	Wet distillers grains with solubles Antimicrobial administration	Rectum	Monensin and Tylosin
Jacob (2010) (39)	Randomized controlled trial	Kansas, United States	Feedlot	Fat cattle preslaughter	Metal supplementation	Rectum	Copper and Zinc
LeJeune (2004) (40)	Cross-sectional study	United States, Multiple States	Retail	Not applicable	“Raised without Antibiotics”	Retail	Not applicable
Muller (2018) (41)	Randomized controlled trial	Kansas, United States	Feedlot	Yearling	Antimicrobial administration	Pen floor	Tylosin
Murray (2020) (42)	Randomized controlled trial	Texas, United States	Feedlot	Yearling	Antimicrobial administration Probiotic supplementation	Unspecified site	Tylosin, *Saccharomyces cerevisiae* and *E. faecium* probiotic
Murray (2021) (43)	Randomized controlled trial	Kansas, United States	Feedlot	Yearling	Metal supplementation Essential oil supplementation	Rectum	Zinc
Murray (2022) (44)	Randomized controlled trial	Texas, United States	Feedlot	Yearling	Antimicrobial administration Probiotic supplementation	Rectum	Tylosin and *Enterococcus faecium* probiotic
Platt (2008) (45)	Randomized controlled trial	Texas, United States	Feedlot	Yearling	Antimicrobial administration	Rectum	Chlortetracycline
Schmidt (2020) (46)	Randomized controlled trial	Nebraska, United States	Feedlot	Fall-placed calves	Antimicrobial administration	Rectum	Tylosin
Schmidt (2021) (47)	Cross-sectional study	United States, Multiple States	Retail	Not applicable	“Raised without Antibiotics”	Retail	Not applicable
Shen (2019) (48)	Randomized controlled trial	Alberta, Canada	Feedlot	Yearling	Antimicrobial administration Probiotic supplementation	Unspecified site	Tylosin, Monensin, *Saccharomyces cerevisiae*
Vikram (2017) (49)	Cross-sectional study	United States, state not indicated	Abattoir	Not applicable	“Raised without Antibiotics”	Post-evisceration	Not applicable
Vikram (2018) (50)	Cross-sectional study	United States, multiple states	Retail	Not applicable	“Raised without Antibiotics”	Retail	Not applicable
Zaheer (2013) (51)	Non-randomized trial	Alberta, Canada	Feedlot	Yearling	Antimicrobial administration	Rectum	Tilmicosin, Tulathromycin, Tylosin
Zhang (2010) (52)	Cross-sectional study	United States, multiple States	Retail	Not applicable	Grass fed	Retail	Not applicable

### 3.2. Study characteristics

**Figure 2:**
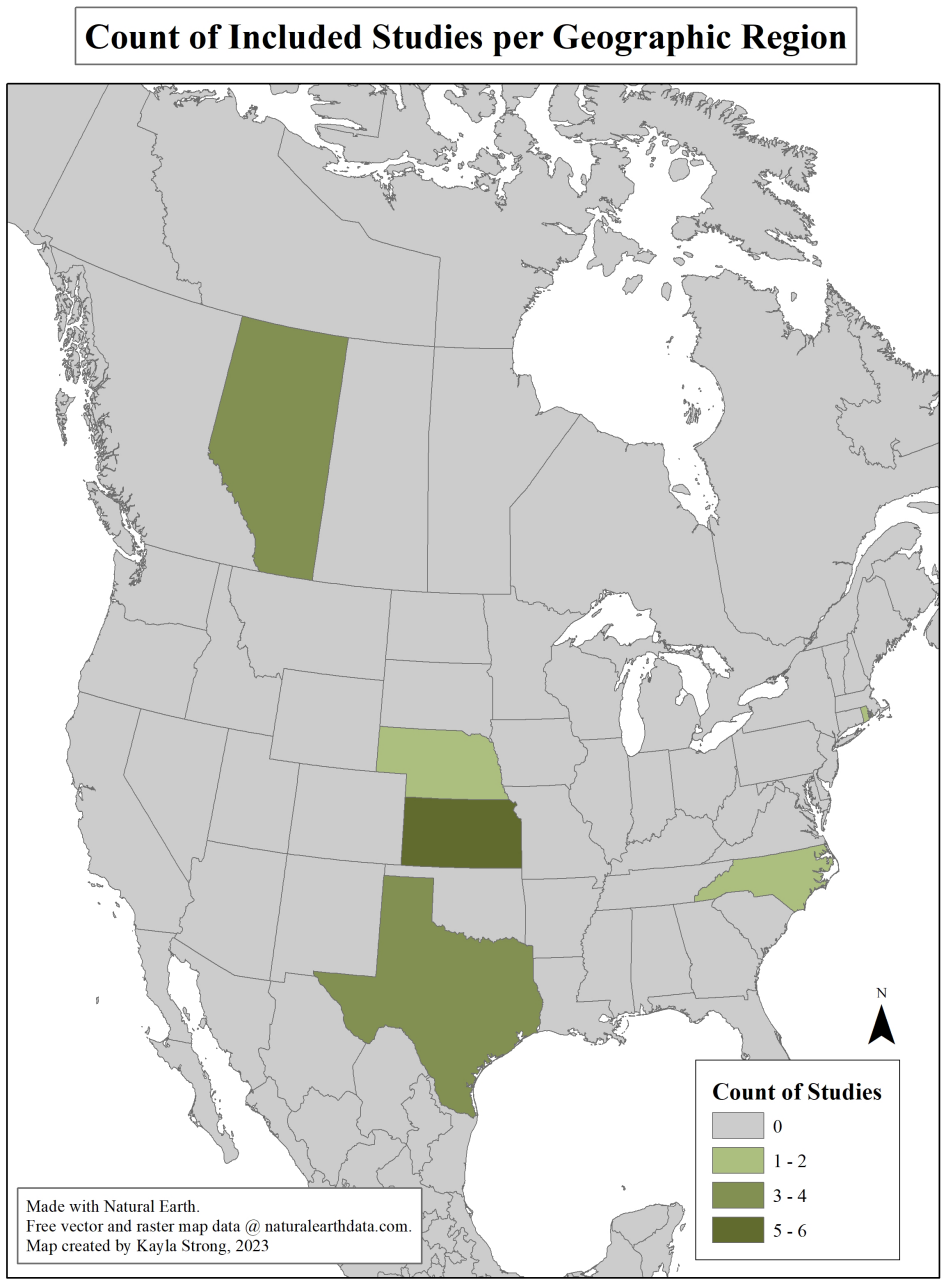
Geographic distribution of articles included in the Scoping review of reported factors associated with antimicrobial resistant enterococci in Canadian beef cattle. Seven articles’ research locations in the United States were not state-specific and are thus not included in the map.

Article attributes are summarized in Supplementary Tables S1, S2. No quality assessment was made regarding the results or interpretation of the articles. Articles published from 2019 to 2022 accounted for 38% (10/26) of included studies. All studies included were conducted in North America, with 22 and four from the United States and Canada, respectively. Geographic distribution is illustrated in Figure 2. International studies examined interventions considered applicable to the Canadian context, having antimicrobial stewardship practices and animal production policies comparable to the Canadian beef production system.

Most studies were done in a feedlot environment (*n* = 17), followed by retail (*n* = 6), abattoir (*n* = 1), and farm (*n* = 2). Enterococci samples came predominately from fecal samples or beef products. Fecal samples were collected either directly from the rectum (*n* = 13), from the pen-floor (*n* = 4), or from an unspecified site (*n* = 2). The remaining samples were collected post-evisceration (*n* = 1) or from retail beef (*n* = 6). Cattle represented in the articles were yearlings (*n* = 10), fall-placed calves (*n* = 5), cows and pre-weaned calves at cow-calf operations (*n* = 2), and finished cattle preslaughter (*n* = 2). Five studies described cattle whose age or weight was not defined; however, these parameters were estimated based on the study context (36, 42–45). The remaining seven studies examined beef samples at retail.

The study design and associated sample collection varied widely across studies. Eight of 17 feedlot studies had cattle acclimatized to the feedlot prior to the study for variable intervals, ranging from 3 days (34, 35) to 3 months (39), whereas study design and trial duration ranged from cross-sectional to cohort studies with longitudinal sampling up to 225 days post-trial initiation (one-day preslaughter) (30). There was also variation in the timing of sample collection compared to the time of intervention. For example, in the 17 feedlot studies, samples were collected during or following the intervention, whereas other studies, at farm, abattoir and retail stages, compared interventions that may have occurred up to several years before sampling. There was also notable variation in the type of feedlot study environments, with five studies reporting on cattle housed in individual pens at an experimental facility, whereas the remaining 12 studies discussed cattle housed in pairs, small groups with 15 or fewer, or in commercial feedlots with more than 100 cattle per pen.

### 3.3. Antimicrobial susceptibility

**Figure 3:**
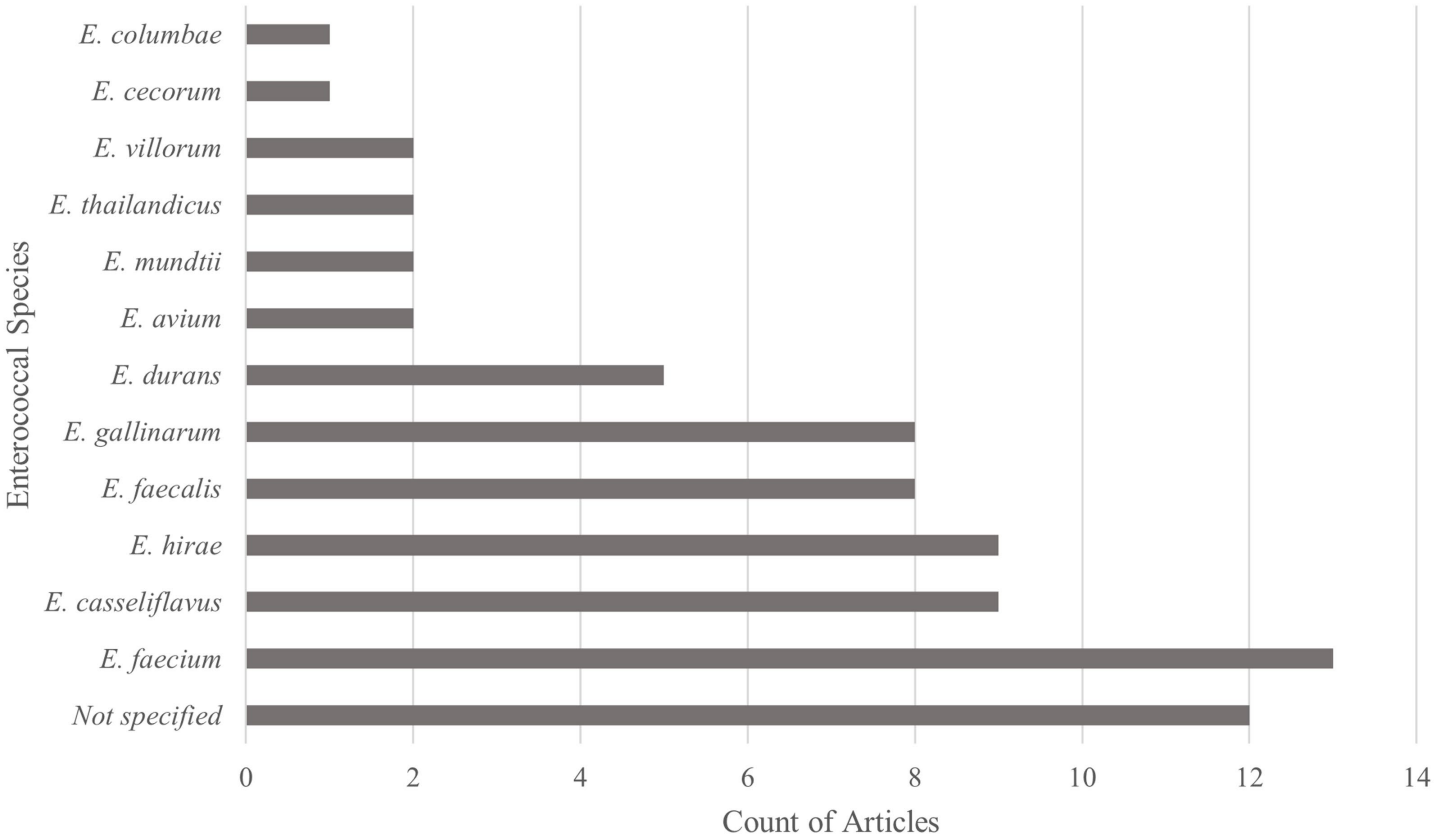
Types of enterococcal species reported within Scoping review of reported factors associated with antimicrobial resistant enterococci in Canadian beef cattle (*n* = 26). Graph reports the number of articles reporting each enterococcal species. Thirteen articles discussed more than one *Enterococcus* species, with a maximum of nine species discussed within one article.

Studies were screened based on the inclusion of *Enterococcus* species-specific findings. Of the 26 included articles, 12 *Enterococcus* species were reported, with *Enterococcus faecium* being the most common, followed by *Enterococcus hirae* and *Enterococcus casseliflavus*. Articles reported one to nine unique *Enterococcus* spp., with a median of four species, whereas 12 articles only reported results to the *Enterococcus* spp. level. The counts of *Enterococcus* species reported are in Figure 3.

Antimicrobial resistance was identified based on phenotypic susceptibility or the presence of AMR genes. AMR in *Enterococcus* spp. isolates were measured phenotypically for 26 studies; and phenotypically and genotypically for 14 studies. Five articles (42, 46, 47, 49, 50) measured genotypic resistance, but outcomes were not reported specific to enterococci but rather the broader sample microbiome using quantitative polymerase chain reaction (PCR) and metagenomic sequencing. An additional three articles reported genotypic resistance specific to enterococci, but were not stratified or statistically evaluated in the comparison of intervention (exposure) or referent groups (30, 32, 51). Similarly, three articles reported phenotypic resistance; however, the resistance findings were not stratified or statistically evaluated in the comparison of intervention (exposure) or referent groups (28, 29, 42) (Supplementary Table S5).

There were diverse methods used to measure antimicrobial susceptibility of enterococci in these studies, including selective media, automated methods (i.e., broth microdilution), and manual methods (i.e., disc diffusion) for phenotypic patterns, whereas PCR and whole genome sequencing were used for genotypic resistance. One study used PCR and whole genome sequencing (49). Of the 26 studies examining phenotypic resistance, 16 cited standardized guidelines for setting interpretive criteria, with multiple methods often described within a single study. If a study stated the use of a specific Sensititre^™^ (Thermofisher Scientific, United States) antimicrobial susceptibility panel, the associated organization’s interpretive guidelines were assumed. Notably, 17 studies used selective, antimicrobial-impregnated media when identifying resistant bacteria. Seventeen studies stated the interpretive criteria or breakpoints used to classify isolates as susceptible, intermediate or resistant in the text, whereas 11 studies stated MICs in the text. The most common guidelines for interpretive breakpoints were referenced from the Clinical and Laboratory Standards Institute (CLSI; *n* = 15), followed by National Antimicrobial Resistance Monitoring System for Enteric Bacteria (NARMS; *n* = 4). In that regard, NARMS describes using CLSI breakpoints when available, and uses their own data to help infer breakpoints when not available (54). In addition, a single study described using a European Committee on Antimicrobial Susceptibility Testing (EUCAST) breakpoint when none was available through CLSI.

### 3.4. Statistics

The hierarchical nature of the data (i.e., multiple isolates per sample, multiple samples per animal, and multiple cattle per pen) often required sophisticated modelling to properly account for potential clustering effects. There was much variation in the types of data analyses used, ranging from descriptive statistics (10) to mixed-effects modelling (16). Sample sizes ranged from 12 (31, 34) to 7576 (32) cattle; however, the studies’ experimental units included individual cattle, pens of cattle, or other aggregated features. Only three studies referenced a sample size calculation or justification for the sample size used (34, 35, 37).

### 3.5. Study findings

Studies often had multiple study questions and objectives, with a wide variety of key findings specific to phenotypic and genotypic resistance (Supplementary Table S3). Outcomes represented AMR in enterococci based on specific genes known to convey antimicrobial resistance or phenotypic antimicrobial susceptibility of isolates (55) at one or more time points. Many studies reported varying temporal associations between AMR outcomes and the timing of the intervention (30, 39, 41, 45, 46, 48).

#### 3.5.1. Factors identified within study findings

Overall, 37 factors were reported from the 26 articles. Nine articles reported multiple factors, with five factors overlapping between studies (i.e., “Raised Without Antibiotics” labelling). Factors were compared between exposed and unexposed groups to assess if they were associated with specific AMR outcomes in *Enterococcus* spp. Factors were broadly summarized based on exposure class, exposure (factor), and whether the article reported a statistically significant association with AMR outcomes in enterococci (Supplementary Table S3). Studies reported associations derived from comparisons between factors and multiple outcomes, such as genotypic and/or phenotypic resistance. These comparisons sometimes resulted in significant associations for one resistance measurement but not another. In addition, some exposures/factors may include multiple exposure groups. For example, some antimicrobial administration studies examined more than one antimicrobial, enabling multiple comparisons to be made to the null when describing that factor.

##### 3.5.1.1. Antimicrobial use

At the genus level, specific to antimicrobial use, studies reported that the use of injectable enrofloxacin (36) or in feed monensin (38) were associated with AMR in enterococci strains. However, other studies reported that injectable formulations of florfenicol (35), danofloxacin (34), or ceftiofur (27) were not associated with AMR in enterococci. One study reported that in-feed virginiamycin use (33) was not associated with phenotypic resistance but with a higher prevalence of identification of the *ermB* gene, associated with resistance to macrolides, lincosamides, and streptogramin B. Reported associations between in-feed macrolide use and resistance were mixed, with inconsistent results across studies and variation between phenotypic and genotypic resistance detection. One study reported an association between macrolide use (both in-feed and injectable) and increased erythromycin resistance in enterococci (51), whereas other studies reported no similar association, specific to in-feed supplementation (32, 41). An association between macrolide feed supplementation and detection of resistance genes *tcr(B)*, associated with copper resistance and *erm(B)* were identified in fecal isolates (29).

##### 3.5.1.2. Production factors

Two studies reported that conventionally raised cattle and beef products were associated with increased resistant enterococci in comparison to those labelled as “Raised Without Antibiotics,” when comparing phenotypic resistance (47, 49). A separate study concluded that these differences were modest and may be linked to product suppliers, based on a significant interaction with the production system (50). Given the time interval and production steps that occurred between factor occurrence (administration of antimicrobials) and time of measurement (retail beef products), several potential confounders may have influenced studies examining the impacts of “Raised Without Antibiotics.” Three studies compared the presence of vancomycin-resistant enterococci (VRE) isolates in conventionally raised beef and beef “Raised Without Antibiotics” or similar labelling, with no evidence of VRE detected in either sample set (31, 40, 47).

The type of processing facility (organic, conventional, or split) was also associated with resistance (37). In a single study that specifically compared grass-fed beef products to conventional beef product, isolates from conventional beef samples were more frequently resistant to daptomycin and linezolid (52). However, other resistance phenotypes assessed were relatively comparable. The study’s authors noted the possibility of the sample including enterococci with intrinsic resistance, given the low resistance to daptomycin and linezolid in most *Enterococcus* spp. (52). Perhaps other studies investigating antimicrobial use labelling of retail meats also involved grass-fed cattle, but this was not explicitly stated in the sample collection strategy, and therefore not considered a grass-fed factor.

##### 3.5.1.3. Other supplements

Antimicrobial resistance was not associated with feeding wet distillers grains with solubles (WDGS), except for flavomycin, where WDGS was associated with decreased frequency of resistance in enterococci isolates (38). Probiotics were also examined, with one study reporting a non-significant trend of decreased antimicrobial resistance when probiotics were used compared to not used (44), whereas another reported no association (48). In one article, supplementing an *Enterococcus faecium*, and *S. cerevisiae*-based probiotic increased the probiotic enterococci sequence type (ST296), with a subsequent decrease in sequence type ST240 that tended to include *erm(B)* and *tet(M)* AMR genes (42). Notably, the probiotic *Enterococcus faecium* strain ST296 was isolated from the manure pack sample 112 days post-trial. The probiotic strain survived drying and milling, simulating the process of manure turning to dust and establishing cyclic transmission of a macrolide-susceptible ST296 strain (slightly altered from the original) within a feedlot (42).

Reported associations related to metal supplementation were mixed and inconsistent across phenotypic and genotypic resistance outcomes for various antimicrobials. The resistance gene *ermB* was reported to be associated (29) or not associated (39) with copper supplementation. Similarly, *tcrB* was reported to be associated with (28, 29) or not identified in either the copper-supplemented or control sample set (39). The resistance gene *tet(M)* was not associated with copper supplementation (29, 39). Phenotypic resistance to chloramphenicol, ciprofloxacin, gentamicin, linezolid, penicillin, streptomycin, vancomycin, zinc, and copper were not associated with copper supplementation (39). Comparably, an association between zinc supplementation and tetracycline resistance was reported (43) but had no other resistance associations (39, 43).

Four articles reported that AMR varied across sampling periods of various study designs, with associations between AMR and AMU during the study but no significant association at the end of the study. These associations were related to the timing of sample collection and timing of antimicrobial treatment, more commonly described in feedlot trials due to study design. Temporary AMR associations were reported following the administration of either chlortetracycline (45), or tylosin (30, 48), but these did not persist and had disappeared by the end of the trials. These factors were all specific to in-feed treatments. Eleven trials included sampling over the entire feeding period or sampling pre-slaughter and were therefore comparable with preslaughter levels, whereas other trials were of shorter intervals, with final sampling dates not representative of the preslaughter period.

## 4. Discussion

This review examined factors associated with AMR in enterococci isolates at all time points along the beef production continuum, from the cow-calf operation to retail markets. Four broad stages of cow-calf, feedlot, abattoir, and retail were identified. Various sub-stages presented opportunities for further research on potential AMR factors. For example, within cow-calf production, there were unique risks associated with neonatal, pasture-grazed, and pre-weaned calves and cows.

Articles within this review addressed a broad range of research objectives and spanned One Health sectors by including articles across the human, animal, and environmental spectrums, with the analysis done by a cross-disciplinary team. A One Health approach supported a robust interpretation of the available information. There were notable variations in study design, antimicrobial susceptibility testing, data analyses and differences in species of enterococci assessed; thus, caution is required when attempting comparisons or summaries of literature. This scoping review described findings but did not try to compare them.

Findings were often temporally specific in that AMR outcomes were often associated with samples collected soon after antimicrobial exposure. This temporal nature of association was not addressed in the study design of many articles, making comparisons among studies difficult. Sampling plans, such as sampling frequency, also varied across the treatment timeline. Three studies reported temporal associations with antimicrobial use which returned to null by the end of the feeding period (30, 45, 48), whereas other studies did not have a study design appropriate to identify this phenomenon. Studies also varied regarding a period of acclimatization to the feedlot for calves before starting a trial; arguably extended acclimatization renders cattle not a “real” feedlot population and therefore less generalizable to feedlot practice. There are concerns that the microbiome may have differed after cattle commingled compared to cattle not given that opportunity, influencing generalizability to other populations. The size of pens which cattle were commingled may also influence the microbiome and reduce comparisons of differing study designs.

AMR detection and reporting varied by specific antimicrobial and *Enterococcus* spp. (33, 45), highlighting the importance of using caution when comparing study findings across all Entercocci. Various species of enterococci may differ in common acquired resistance patterns and intrinsic resistance. For example, *Enterococcus faecalis* is intrinsically resistant to quinupristin-dalfopristin, whereas *Enterococcus faecium* is not (56). Phenotypic erythromycin and tetracycline resistance, and resistance genes specific to tetracyclines (*tet(M)*) and macrolides (*erm(B)*) were the most common resistance trends identified in enterococci. These were consistent with a prior enterococci-specific review and a surveillance study of AMR in isolates from various stages of beef production (2, 22). Zaheer et al. (2) reported that various enterococci species were highly associated with their environment. Specifically, *Enterococcus hirae* was predominant within feedlot cattle settings yet accounted for < 15% of enterococci in beef processing systems, abattoirs, and retail spaces (2). Instead, *Enterococcus faecalis* was the most predominant enterococcus species in abattoirs and retail spaces, accounting for 74% of samples (2). The predominance of *Enterococcus faecalis* in abattoirs and retail spaces has been seen in additional studies (57–59). Human clinical isolates are primarily *Enterococcus faecalis* (2), the predominant concern in human medicine (12, 60). The relevance of *Enterococcus hirae* for humans is not fully understood as it is rarely recognized in humans, although it may not always be identified due to the limitations of some commercial diagnostic identification methods (61). The shift from *Enterococcus hirae* predominance in cattle to *Enterococcus faecalis* in the abattoir and retail beef did not provide evidence of transmission across the continuum. Furthermore, this observation highlighted the importance of speciating enterococci when evaluating factors that might be associated with AMR. Individual enterococci species were reported in 14 of 26 studies assessed within this review. Twelve studies just reported *Enterococcus* spp., which is also a concern considering intrinsic resistance differs among species.

Several studies investigated associations between a high level of copper supplemented in the feed and the presence of *tcrB*, a transmissible gene conferring resistance to copper, in enterococci isolated from the feces of those cattle compared to enterococci that were isolated from feces of cattle supplemented at a lower concentration covering dietary needs for cattle. The *tcrB* gene has been previously identified co-located on mobile genetic elements that also carry *erm(B)*, a gene that encodes resistance to macrolides, lincosamides, and streptogramin B, and/or tet(M), a gene that encodes resistance to tetracyclines (62, 63).

### 4.1. Multicausal associations

Articles within this review discussed the long-term and multi-factorial nature of AMR (27, 42, 44). The concept of a multicausal association was further illustrated when considering the number of studies that examined exposures that potentially occurred months or years before sampling, for example, beef products raised conventionally versus those “Raised Without Antibiotics” or similar labelling (31, 37, 40, 47, 50), grass-fed (52), or studies attempting to assess effects of antimicrobial supplementation that had occurred years earlier (27). In the example of “Raised Without Antibiotics” versus conventional beef production, it is difficult to conclude if the reported associations (or lack of) resulted from antimicrobial exposure or were related to other various production, transportation, abattoir, processing, or retail exposures. Differing constellations of factors and confounders in long-term studies may not be measured or adjusted for in statistical analyses.

Some feedlot studies reported a similar increase in the proportion of resistant isolates in both the control and intervention group earlier in the feeding period and a similar decrease in the proportion of resistant isolates over time. In addition to changes in diet and microbiome mentioned above, perhaps there was an additional environmental transmission of bacteria and/or their resistance genes between the groups in the feedlot over time. For example, Beukers et al. (30) followed the proportion of tylosin resistance fecal enterococci isolates in cattle receiving tylosin phosphate versus those in control cattle. Despite a difference in the frequency of resistance across isolates, there was a similar distribution within both treatment groups, with parallel timing of increases and decreases of the proportion of resistance (30).

### 4.2. Multilevel data and issues of clustering

Articles included within this scoping review varied widely in sample collection methodologies (i.e., individual versus composite samples) and the experimental unit studied. In many studies, the exposure unit was not the same as the unit of measurement. For example, individual cattle received antimicrobial treatment, but resistance was assessed in *Enterococcus* spp. isolated from pooled fecal samples. Many but not all studies accounted for this multilevel data structure in their statistical analysis using mixed-effects models. Articles that reported adjusted data accounting for its hierarchical structure when discussing significance rarely maintained the hierarchical structure of data within result summaries or supplemental material. Many studies did not reflect the results of these analyses with any level of detail. When data does not present the sampling structure, future use of raw data may introduce clustering biases and misrepresent the data. Going forward, publishers should encourage data to be presented at all appropriate levels when presenting summaries of results and within their supplemental material. In addition, authors should provide details of the stochastic methods of analysis and subsequent interpretation of their findings to promote reproducibility.

### 4.3. Standardization

Increased standardization and reproducibility of existing research studies would be extremely valuable for strengthening current knowledge in AMR. The earliest published articles included in the review were published in 2004 (40), and standardization of reporting has subsequently evolved. This was evidenced through updated reporting standards and guidelines that have been expanded to account for trial protocol accessibility in randomized trials (64), and developed to address the needs of observational epidemiological studies (65). Despite these advances, there remains wide variation in data presented in articles published in the past 5 years, indicating standardization has not been achieved. This might include further harmonization across national standards, more robust reporting guidelines by journals, or incentivization to provide anonymized hierarchical data and model parameters.

A recent systematic review with a narrower focus on macrolide supplementation in the feedlot setting concluded that long durations of tylosin supplementation are associated with increased proportions of macrolide-resistant gastrointestinal enterococci in feedlot cattle (22). The review encouraged researchers to follow reporting guidelines and publish comparison data for a meta-analysis (22), consistent with the challenges faced in this scoping review.

### 4.4. Knowledge gaps

This review examined factors occurring within four core stages in beef production: cow-calf operations, feedlot, abattoir, and retail. Within each stage, a series of substages or categorizations were attempted (i.e., neonatal, pasture-grazed, and pre-weaned calves). Of the 26 articles included, only two (27, 36) examined exposures at the cow-calf and “farm” space, making it difficult to differentiate risks across sub-stages.

The feedlot was the second identified stage, where cattle typically spend 90 to >300 days. Most studies identified within the scoping review occurred within the feedlot environment. However, there were knowledge gaps along the temporal timeline, with few studies examining cattle for the total duration at the feedlot. Reproducibility and replication of studies in a comparable environment with similar sampling timelines were limited, presenting an additional knowledge gap. Many feedlot studies occurred in an experimental pen setting, with individual animals or small groups from single sources, and may have included an acclimatization period. In contrast, commercial feedlot settings in North America are often much larger and introduce cattle from numerous sources. Therefore, findings from experimental pen settings may not be generalizable to the commercial environment given multiple potential confounders that may occur in commercial feedlots. This introduces a knowledge gap when interpreting these experimental pen studies.

After the feedlot phase, cattle are transported to the abattoir for slaughter and processing. Similar to the prior two stages, a series of sub-stages occur. These include transport to slaughter, lairage, slaughter, processing, and secondary processing as required. Within the articles identified, only one study applied to these stages, examining abattoir factors (37).

The final stage was the retail space, which includes packaging, storage, transport, potential repackaging, and purchasing conditions of the meat. Although multiple studies examined resistance at the retail stage, the exposure in question was the use of antimicrobials in raising cattle. However, there were no studies specifically examining retail interventions/exposures. Further research and discussion of potential AMR-related factors related to cow-calf operations, transportation, abattoirs, beef processing, and retail spaces are required.

Parallel to knowledge gaps in the scope of research, there are also potential gaps in the depth of information. A recent review discussed the benefits of whole genome sequencing in detecting AMR genes in enterococci and concluded that this approach is well-suited for identifying phenotypically sensitive bacteria that may carry resistance genes (66). Identification of genetic determinants allows for potential outbreak management and understanding of the potential for phylogeographic spread, enhancing understanding of AMR epidemiology (67). Genotypic data regarding potential factors associated with AMR are currently limited and represent a substantial data gap in the literature.

### 4.5. Limitations

Several limitations may have affected the type of articles retrieved and included in this scoping review. First, articles were not excluded based on the quality of the evidence. A minimal number of publications met the search criteria, and we wanted to characterize all available information, make interpretations, and suggest future actions. The quality of evidence was assessed internally but was not reflected as a part of this scoping review. Secondly, environmental articles were not included. Environmental transmission is an essential component of AMR within beef production but was outside the context of this scoping review. Thirdly, grey literature (e.g., conference proceedings, dissertations, government publications) was not captured within the scoping review. This potentially excluded smaller studies and emerging, unpublished research. Additionally, only articles written in English were included, potentially excluding international findings applicable to the Canadian context. The requirement of there being a comparison group for inclusion of a factor excluded certain study designs, e.g., descriptive studies and case reports.

The extraction of factors associated with AMR in enterococcal isolates from articles included in the scoping review was unique to this review and identified challenges in data extraction for secondary purposes. Factors were drawn from reported associations and patterns; however, summarized statements were unique to the context of the study and often not comparable to other studies examining the same factor. This was due to differences in sampling timelines, antimicrobial susceptibility testing protocols, type of data presented, and confounding variables considered. In addition, methods of bacterial analysis, antimicrobial susceptibility testing, and even minimum inhibitory concentrations and breakpoint cut-offs have changed over time. Therefore, caution must be used when interpreting findings and drawing conclusions beyond the scope of the original article. The differences in articles examining similar factors limit opportunities for meta-analysis and other quantitative analyses.

Results from the data extraction were not presented specific to each enterococcal species, and instead discussed as a collective genus. The decision to report at the genus level was due to variations in detail provided by the articles, with 14 articles providing species of Enterococci and 12 not. Of those who did report the enterococci species, there were varying speciation methodologies and standards used. The decision not to report enterococci species was a limitation in this article given the intrinsic resistance trends that are unique to many Enterococci species, and differing environments in which species are detected.

The scoping review faced similar challenges as prior antimicrobial-specific reviews in the area, with limited articles for inclusion, variable study designs, limited data available for extraction, inadequate adjustments for potential confounders, and reporting of non-significant results by omission, potentially furthering publication bias (22, 68, 69). A general limitation of scoping reviews is the possibility that the search strategy did not identify all published articles within the study scope; however, this risk was minimized by having a multidisciplinary team involved in syntax development and study design.

## 5. Conclusion

This scoping review identified factors that may be associated with increases or decreases in the prevalence of AMR in *Enterococcus* spp. isolated at various points along the beef production continuum, including cow-calf and feedlot operations, slaughter, and retail markets. A series of factors associated with antimicrobial administration, metal supplementation, probiotics supplementation, and meat processing were characterized. Resistance was associated with certain heavy metals and antimicrobial supplementation but was highly specific to the timing of sampling related to exposure, and specific phenotypic and/or genotypic resistance assessed. Inconsistencies in the amount of detail, availability of reported results, and interpretation of hierarchical data limited the interpretability and comparison of factors on a broader One-Health scope. Data gaps were identified in antimicrobial treatment and other management factors occurring during breeding, neonatal environment, and pasture grazing stages at cow-calf operations; transportation between production stages; abattoir lairage, slaughter, processing, and potential secondary processing; and packaging, storage and purchasing conditions in retail environments. Variations in sampling methods, sampling framework, intervention/exposure timeline and duration, data presentation, and resistance information collected were additional limitations. Future research should focus on filling identified research gaps that have limited or no published articles, along with standardization of laboratory, analytical and reporting methodologies. In addition, manuscripts should prioritize access to anonymized raw data with associated metadata for secondary analyses for future transdisciplinary projects and applications.

## Data availability statement

The original contributions presented in the study are included in the article/Supplementary material, further inquiries can be directed to the corresponding author.

## Author contributions

KS, RR-S, CW, SO, and SC: conceptualization. KS, RR-S, SO, SC, and HG: methodology. KS, KM, JI, and SC: investigation. KS, KM, JI, and HG: data curation. KS: writing—original draft. KS, RR-S, CW, SO, KM, JI, JK, SC, and HG: writing—review and editing. RR-S and SC: supervision and funding acquisition. All authors contributed to the article and approved the submitted version.

## Funding

This research is part of the AMR—One Health Consortium, funded by the Major Innovation Fund program of the Ministry of Jobs, Economy and Innovation, Government of Alberta. This project is also funded by the Natural Sciences and Engineering Research Council of Canada and the Genomics Research Development Initiative Shared Priority Project on AMR (GRDI-AMR 1).

## Conflict of interest

The authors declare that the research was conducted in the absence of any commercial or financial relationships that could be construed as a potential conflict of interest.

## Publisher’s note

All claims expressed in this article are solely those of the authors and do not necessarily represent those of their affiliated organizations, or those of the publisher, the editors and the reviewers. Any product that may be evaluated in this article, or claim that may be made by its manufacturer, is not guaranteed or endorsed by the publisher.
