# Supplementary material for: Factors associated with antimicrobial resistant enterococci in Canadian beef cattle: A scoping review
Source: Front Vet Sci. 2023 Apr 20;10:1155772. doi: 10.3389/fvets.2023.1155772 (PMC10157153; doi:10.3389/fvets.2023.1155772)
Supplement: Supplementary file 1 [file Data_Sheet_1.pdf]

Supplementary Material

Factors associated with antimicrobial resistant enterococci in Canadian beef cattle: a scoping review

Kayla M. Strong*, Kaitlin L. Marasco, Jesse Invik, Heather Ganshorn, Richard J. Reid-Smith, Cheryl L. Waldner, Simon J. G. Otto, John P. Kastelic, Sylvia L. Checkley

*** Correspondence:** Kayla Strong: kayla.strong@ucalgary.ca

Table of Contents

S1. Search strategy 2

S2. Screening inclusion and exclusion reviewer manual 6

S3. Data extraction manual 8

S4. Summary of study attributes of included scoping review articles. 12

S5. Descriptive details from included scoping review articles. 14

S6. Enterococci resistance factors identified from scoping review articles. 15

List of Tables

Table S1: Summary of articles included in the scoping review of reported factors associated with antimicrobial resistant enterococci in Canadian beef cattle categorized by study attributes. 12

Table S2: Descriptive details of articles included in the scoping review of reported factors associated with antimicrobial resistant enterococci in Canadian beef cattle. 14

Table S3: Enterococci resistance factors identified from articles included in the scoping review of reported factors associated with antimicrobial-resistant enterococci in Canadian beef cattle. 15

List of Figures

Figure S1: CAB Abstracts Search Strategy for articles considered for inclusion in the scoping review of reported factors associated with antimicrobial resistant enterococci in Canadian beef cattle. Page 1 3

Figure S2: CAB Abstracts Search Strategy for articles considered for inclusion in the scoping review of reported factors associated with antimicrobial resistant enterococci in Canadian beef cattle. Page 2 4

Figure S3: CAB Abstracts Search Strategy for articles considered for inclusion in the scoping review of reported factors associated with antimicrobial resistant enterococci in Canadian beef cattle. Page 3 5

S1. Search strategy

The following is an example search strategy for articles considered for inclusion in the scoping review of reported factors associated with antimicrobial resistant enterococci in Canadian beef cattle. CAB Abstracts Search Strategy is used as an example.

MY

EBSCOhost

Friday, June 04, 2021 9:42:33 PM

#	Query	Limiters/Expanders	Last Run Via	Results
S1	DE "cattle" OR DE "beef cattle" OR DE "beef herds" OR DE "suckler herds" OR DE "bulls" OR DE "calves" OR DE "cows" OR DE "heifers" OR DE "steers" OR DE "meat production" OR DE "beef production" OR DE "meat and livestock industry"	Search modes - Find all my search terms	Interface - EBSCOhost Research Databases Search Screen - Advanced Search Database - CAB Abstracts	537,103
S2	TI ((beef OR suckler*) AND (cattle OR cow* OR heifer* OR steer* OR stocker* OR bull* OR feeder* OR "cow-calf")) OR AB ((beef OR suckler*) AND (cattle OR cow* OR heifer* OR steer* OR stocker* OR bull* OR feeder* OR "cow-calf"))	Search modes - Find all my search terms	Interface - EBSCOhost Research Databases Search Screen - Advanced Search Database - CAB Abstracts	39,417
S3	TI ((retail OR butcher* OR grocer* OR supermarket*) AND (beef OR meat)) OR AB ((retail OR butcher* OR grocer* OR supermarket*) AND (beef OR meat))	Search modes - Find all my search terms	Interface - EBSCOhost Research Databases Search Screen - Advanced Search Database - CAB Abstracts	6,145
S4	S1 OR S2 OR S3	Search modes - Find all my search terms	Interface - EBSCOhost Research Databases Search Screen - Advanced Search Database - CAB Abstracts	544,879
S5	((DE "drug resistance" OR DE "multiple drug resistance") AND (DE "antibiotics" OR DE	Search modes - SmartText Searching	Interface - EBSCOhost Research Databases Search Screen - Advanced	14,088

Figure S1: CAB Abstracts Search Strategy for articles considered for inclusion in the scoping review of reported factors associated with antimicrobial resistant enterococci in Canadian beef cattle. Page 1

	"antiinfective agents" OR DE "antibacterial agents"))		Search<br>Database - CAB Abstracts	
S6	TI ((antimicrobial* OR antibiotic* OR "anti biotic*"OR multidrug* or "multidrug" OR microbial* ORdrug* OR antibacterial* OR bacteria* OR "multiple drug*" OR multiclass OR "multi class" OR "multiple class") n3 (resistan* or susceptib*)) OR AB ((antimicrobial* OR antibiotic* OR "anti biotic*"OR multidrug* or "multidrug" OR microbial* ORdrug* OR antibacterial* OR bacteria* OR "multiple drug*" OR multiclass OR "multi class" OR "multiple class") n3 (resistan* or susceptib*))	Search modes - Find all my search terms	Interface - EBSCOhost Research Databases<br>Search Screen - Advanced Search<br>Database - CAB Abstracts	63,604
S7	S5 OR S6	Search modes - Find all my search terms	Interface - EBSCOhost Research Databases<br>Search Screen - Advanced Search<br>Database - CAB Abstracts	65,601
S8	DE "Enterococcus" OR DE "Enterococcus faecalis" OR DE "Enterococcus faecium" OR DE "Enterococcus hirae"	Search modes - Find all my search terms	Interface - EBSCOhost Research Databases<br>Search Screen - Advanced Search<br>Database - CAB Abstracts	17,615
S9	TI (Enterococc* or "e. faecalis" or "e. faecium" or "e. hirae") OR AB (Enterococc* or "e. faecalis" or "e. faecium" or "e. hirae")	Search modes - Find all my search terms	Interface - EBSCOhost Research Databases<br>Search Screen - Advanced Search<br>Database - CAB Abstracts	17,565
S10	S8 OR S9	Search modes - Find all my search terms	Interface - EBSCOhost Research Databases<br>Search Screen - Advanced	20,334

Figure S2: CAB Abstracts Search Strategy for articles considered for inclusion in the scoping review of reported factors associated with antimicrobial resistant enterococci in Canadian beef cattle. Page 2

S11	S4 AND S7 AND S10	Search modes - Find all my search terms	Search<br>Database - CAB Abstracts<br><br>Interface - EBSCOhost Research Databases<br>Search Screen - Advanced Search<br>Database - CAB Abstracts	338

Figure S3: CAB Abstracts Search Strategy for articles considered for inclusion in the scoping review of reported factors associated with antimicrobial resistant enterococci in Canadian beef cattle. Page 3

S2. Screening inclusion and exclusion reviewer manual

The following manual was developed to guide screening of articles considered for inclusion in the scoping review of reported factors associated with antimicrobial resistant enterococci in Canadian beef cattle:

Screening inclusion and exclusion reviewer manual

Title and Abstract Screening

- Is the article published before 1984?
  - If yes, exclude. If no, proceed to next question.
- Is the article published in a journal specific to non-beef cattle (i.e., poultry science)?
  - If yes, exclude. If no, proceed to next question.
- Is the article written in a language other than English?
  - If yes, exclude. If no, proceed to next question.
- Is the article a conference abstract, review, or opinion piece?
  - If no, proceed to next question. If yes, exclude.
- Does the article title and abstract include the following terms: Enterococcus, Antimicrobial Resistance, Beef Cattle. Linguistic variants of the term will also be accepted. For example, "Beef Cow," "Beef Heifer," "Beef Steer," and "Beef Calf" are all appropriate alternatives to "Beef Cattle".
  - If no, exclude. If yes, proceed to the next question.
- Is the article using cattle specimens for in vitro enterococci resistance testing?
  - If yes, exclude. If no, include article for Full Text Screening.

Full Text Screening

- Is the article full body text written in English?
  - If yes, proceed to next question. If no, exclude with the following tag: "Article not in English".
- Is the article a conference abstract, review, or opinion piece?
  - If no, proceed to next question. If yes, exclude with the following tag: "Conference Abstract, Review, Opinion Piece".
- Is the article specific to beef cattle or beef products?
  - If yes, proceed to the next question. If no, exclude with the following tag: "Article is not specific to beef cattle or beef products".
- Is the article specific to antimicrobial resistance and *Enterococcus* spp.?
  - If yes, proceed to the next question. If no, exclude with the following tag: "Article not specific to Antimicrobial Resistance and Enterococci".

- Does the article include measurements of antimicrobial resistance between two or more sample groups which have a practice or circumstance influencing the prevalence of AMR Enterococci?[1]
  - If yes, proceed to the next question. If no, exclude with the following tag: "No intervention described and measured".
- Is the practice or circumstances described in the article applicable to the Canadian context?
  - If yes, include the article. If no, exclude with the following tag: "Not applicable to the Canadian context".

---

[1] The iAM.AMR project documentation defines a factor as: A practice or circumstance which influences the occurrence of AMR. This is an intentionally broad definition that does not consider the concept of causality; we consider any relationship between an exposure and outcome as a factor, whether or not we can elucidate a causal pathway."

(iAM.AMR Team. Literature Search [Internet]. iAM.AMR. 2021. Available from: https://docs.iam.amr.pub/en/latest/data_extraction/search.html#what-is-a-factor)

S3. Data extraction manual

The following manual was developed to guide the extraction of information from articles included in the scoping review of reported factors associated with antimicrobial resistant enterococci in Canadian beef cattle:

<u>Included articles data extraction manual</u>

Please extract the following sections into the project Covidence form. If information is not available within the article, please type NA. Please do not leave any cells empty.

<u>Author(s)</u>

- The field will be automatically populated from the RIS file - Please confirm its completeness.

<u>Year of Publication</u>

- The field will be automatically populated from the RIS file - Please confirm its completeness.

<u>Country and State/Province of Origin</u>

- Please identify the country and state or province where the study was conducted.
  - If there are multiple countries, provinces or states involved, please list all.

<u>Design Type</u>

- Please select the appropriate design type from a pre-defined drop-down list.
- A description of each study type is described below:
  - Case Study: An Observational study following a specific subject, group, or case in detail over time.
  - Cross-Sectional Study: An observational study that takes information or measurements from a sample population at a single point in time.
  - Cohort Study: An observational longitudinal study that follows a group of subjects with similar or shared characteristics over time.
  - Case-Control Study: An observational study where subjects with different health outcomes are compared to examine potential differing exposures.
  - Randomized Control Trials: An experimental study where subjects are randomly assigned to differing interventions (often experimental and control groups). A measured response is evaluated for each group following a set time.
  - Non-Randomized Trials: An experimental study where subjects are assigned to differing interventions (often an experimental group and a control group) in a non-random format. A measured response is evaluated for each group following a set time.

<u>Research Aims</u>

- Write the research question the authors intend to answer through the study or the aim of the study.
  - This may be provided in the abstract or in the final paragraph in the introduction.

Study Sample Population

- Where are the samples being taken from?

If Applicable, Source of Cattle and Supplementation Background

- Where did the cattle originate from?
    - For example, are cattle from a university ranch or a cow-calf operation?
- What are their prior antimicrobial and feed supplementation?
    - For example, were cattle given antimicrobial supplementation before the study?
    - If available, provide supplement name and dose.

Sample size

- How many samples are considered in the study?
- If the study occurred in a cow-calf or feedlot setting, state:
    - how many cattle were included
    - how many pens were included
    - how many feedlots were included (if applicable)
    - how many ranches were included (if applicable)

Sampling Methodology

- How were samples collected?

Enterococcus spp.

- What species of *Enterococcus* spp. is being studied?
    - If multiple species are considered, please list all species.
    - If the species is not specified, please state "Not Specified."

Bacteria Isolation Method

- What method was taken to obtain pure bacterial cultures?
    - Methods can be broadly grouped as:
        - Streaking on agar
        - Plate pouring

Antimicrobial Susceptibility Testing

- What method is taken to test the drug resistance in the bacteria?
    - Methods can be broadly grouped as:
        - Broth microdilution
        - Agar dilution
        - Disk diffusion (Kirby-Bauer)
        - Gradient diffusion methods

Explicit Breakpoints

- Breakpoints are the concentration of antimicrobial required to treat bacteria.
- Please enter yes, no, or NA.

<u>If applicable, is this breakpoint consistent with current CIPARS, NARMS or EUCAST breakpoints?</u>

- Please enter yes, no, or NA.

<u>Does the study identify the minimum inhibitory concentration?</u>

- A minimum inhibitory concentration (MIC) is the lowest concentration of an antimicrobial, inhibiting the growth of a strain of bacteria. The information is often provided in a table format.
- Please enter yes, no, or NA.

<u>Study Intervention?</u>

- What intervention is being studied in the article?
    - If multiple interventions are being considered, please list all.

<u>Describe Intervention used in Experimental Group.</u>

- If the article is examining an antimicrobial or supplement intervention:
    - What is the name of the drug?
    - What is the dose of the drug?
    - What is the type of drug?
        - (i.e., Tylosin, 8 mg per pound of bodyweight, macrolide)

<u>Describe Intervention used in Control Group.</u>

- If the article is examining an antimicrobial or supplement intervention:
    - What is the name of the drug?
    - What is the dose of the drug?
    - What is the type of drug?
        - (i.e., Tylosin, 8 mg per pound of bodyweight, macrolide)

<u>Duration of the Intervention (if applicable)</u>

- State the number of days that the intervention is implemented.

<u>Antimicrobial Resistance (AMR) Genes</u>

- State the *Enterococcus* spp. AMR genes measured within the article.

<u>AMR Gene Patterns (Multiple Resistance Reported)</u>

- State the gene patterns reported in the article.
    - Gene patterns are when there is multidrug resistance, in which genes are identified together. An example of a gene pattern is: tcrB + erm(B) + tet(M).

<u>AMR Phenotypes</u>

- State the *Enterococcus.* spp. AMR phenotypes measured within the article.

<u>AMR Phenotypes Patterns (Multiple Resistance Reported)</u>

- State the phenotype patterns reported in the article.
    - Phenotype patterns are when there is multidrug resistance, in which phenotype resistances are identified together. An example of a phenotype pattern is: Vancomycin and erythromycin resistance (VAN + ERY).

<u>Key findings that relate to the scoping review question/s</u>

- Please state the key findings of the paper.

<u>Usable Data for CEDARS</u>

- Please enter yes or no.
- To be included in CEDAR, articles must have:
    - An extractable intervention referred to as a "factor" associated with AMR *Enterococcus* spp.[2]
    - Data must be presented as an odds ratio or prevalence comparison (in text or graph) specific to the "intervention" and "control."
    - The study must use non-selective media.
    - The study must have the total (N) provided for the Intervention and control.

<u>Notes</u>

- Please include any additional notes which are applicable to study evaluation and interpretation.

---

[2] The iAM.AMR project adopts a broad definition when discussing factors and does not apply any causal relation to the term. As per project documentation: "In the context of the IAM.AMR project, we have defined a 'factor' as a practice or circumstance which influences the occurrence of AMR. This is an intentionally broad definition that does not consider the concept of causality; we consider any relationship between an exposure and outcome as a factor, whether or not we can elucidate a causal pathway" (1)

S4. Summary of study attributes of included scoping review articles.

The following table summarizes attributes of articles included in the scoping review, of reported factors associated with antimicrobial resistant enterococci in Canadian beef cattle:

Table S1: Summary of articles included in the scoping review of reported factors associated with antimicrobial resistant enterococci in Canadian beef cattle categorized by study attributes.

Study Attribute			Count	Percentage
**Country**				
	Canada		4	15%
		Alberta	*4*	*100%*
	United States		22	85%
		Kansas	*6*	*27%*
		Multiple	*6*	*27%*
		Nebraska	*2*	*9%*
		North Carolina	*2*	*9%*
		Not Stated	*1*	*5%*
		Rhode Island	*1*	*5%*
		Texas	*4*	*18%*
**Year of Publication**				
	2000 – 2005		2	8%
	2006 – 2010		5	19%
	2011 – 2015		5	19%
	2016 – 2020		8	31%
	>2020		6	23%
**Study Design**				
	Cross-sectional study		9	35%
	Non-randomized trial		3	11%
	Randomised controlled trial		14	54%
**Stage of Sample Collection**				
	Abattoir		1	4%
	Farm		2	8%
	Feedlot		17	65%
	Retail		6	23%

**Exposure Studied**[3]			
	Antimicrobial administration[4]	16	43%
	Macrolides	*10*	
	Fluoroquinolones	*2*	
	3rd Gen Cephalosporins	*1*	
	Amphenicols	*1*	
	Ionophores	*2*	
	Streptogramin	*1*	
	Tetracycline	*1*	
	Raised without antimicrobials	6	16%
	Metal supplementation	4	10%
	Probiotic supplementation	3	8%
	Pen Environment	2	5%
	Wet distillers grains with solubles	1	3%
	Therapeutic versus subtherapeutic antimicrobial use	1	3%
	Grass fed	1	3%
	Processing plant type	1	3%
	Essential oil supplementation	1	3%
	Nutritional supplement	1	3%

[3] Nine articles reported two or more exposures, resulting in 37 factors from 26 articles

[4] Two articles examined two unique antimicrobial classes. Each antimicrobial is listed independently, resulting in a value greater than the count of antimicrobial interventions assessed.

S5. Descriptive details from included scoping review articles.

The following table lists descriptive details from articles included in the scoping review of reported factors associated with antimicrobial resistant enterococci in Canadian beef cattle:

Table S2: Descriptive details of articles included in the scoping review of reported factors associated with antimicrobial resistant enterococci in Canadian beef cattle.

Table provided in supplementary materials excel spreadsheet.

S6. Enterococci resistance factors identified from scoping review articles.

The following table lists Enterococci resistance factors identified from articles included in the scoping review of reported factors associated with antimicrobial-resistant enterococci in Canadian beef cattle. Multiple comparisons may be made within each factor. Association is determined by a significant statistical difference between the intervention (exposure) group and referent group.

Table S3: Enterococci resistance factors identified from articles included in the scoping review of reported factors associated with antimicrobial-resistant enterococci in Canadian beef cattle.

Table provided in supplementary materials excel spreadsheet.
